# Transpulmonary chemoembolization and microwave ablation for recurrent or advanced non-small cell Lung Cancer

**DOI:** 10.1038/s41598-024-76323-y

**Published:** 2024-10-26

**Authors:** Thomas J. Vogl, Kuei-An Chen, Hao Li, Mohamed E.M. Fouad, Zahra Afraz, Hamzah Adwan

**Affiliations:** 1 Clinic for Radiology and Nuclear Medicine, University Hospital Frankfurt, Goethe University, Theodor-Stern-Kai 7, 60590 Frankfurt, Germany; 2 Department of Medical Imaging and Intervention, College of Medicine, Chang Gung Memorial Hospital-Linkou, Chang Gung University, Taoyuan, Taiwan

**Keywords:** Transpulmonary chemoembolization, Microwave ablation, Non-small cell lung cancer, Interventional radiology, Targeted therapies, Cancer imaging, Cancer therapy, Oncology, Lung cancer, Non-small-cell lung cancer

## Abstract

To verify the treatment effect of the combination of transpulmonary chemoembolization (TPCE) and microwave ablation (MWA), targeting the treatment of recurrent or advanced non-small cell lung cancer (NSCLC). A total of 53 patients were studied and grouped according to the diameter of the largest pulmonary nodule, defined as index tumor size (ITS). Patients with an ITS > 3 cm (n = 20) were treated with TPCE and MWA. Patients with an ITS ≤ 3 cm were treated either with a combination therapy (n = 24) or MWA alone (n = 9). The treatment response, including complications and survival outcome, was then analyzed. After TPCE, there was an average ITS reduction of 0.91 cm, and 25% of patients in ITS > 3 cm were downgraded to ITS ≤ 3 cm. After TPCE, there were 12 patients (27%) with PR status and 32 (73%) with SD status. No PD patient in our case series was noted before MWA.The complication rate of MWA was significantly higher in ITS ≤ 3 cm than in ITS > 3 cm (p = 0.013). The median survival time (MST) was 26.7 months, and the time to progression was 13.2 months. The patients in the ITS ≤ 3 cm had longer MST than the others (31.6 vs. 15.8 months, p = 0.003). The significant prognostic factor was ITS > 3 cm (HR: 1.18, p = 0.02). A combination of TPCE and MWA might be feasible to control non-operable, recurrent, or advanced NSCLC.

## Introduction

Primary lung cancer is the second most diagnosed cancer and the leading cause of cancer death^1^. The recommended therapies for primary lung cancer include local resection, such as surgery and stereotactic ablative radiotherapy (SABR), or targeting the tumor progress using systemic chemotherapy, and immunotherapy^2^. However, alternative therapy options are still needed for patient’ s ineligible for surgical resection^3^, radiation^4^, or systemic treatments^2^.

Image-guided lung tumor thermal ablation, including radiofrequency ablation (RFA), microwave ablation (MWA), and cryoablation, were rapidly emerging methods that could be offered as an alternative option for lung tumors. The therapeutic outcome of these methods on oligo-pulmonary metastasis has been reported previously, especially for metastatic tumors known to be radioresistant^3,4^. Thermal ablation could restrict tumor progress and be a curative treatment option in certain conditions^5^ for primary lung cancer, especially non-small cell lung cancer (NSCLC). Thermal ablation was recommended for patients with contraindications to surgery and SABR, patients with multifocal IA NSCLC, or patients suffering from advanced NSCLC with disease progression on target therapy or immunotherapy^6^. Transpulmonary chemoembolization (TPCE) enables selective cannulation of the tumor-supplying pulmonary arteries and targeted infusion with chemotherapeutic medications, lipiodol, and particulate embolization^7^. Pohlen et al. addressed the feasibility, absence of long-term systemic toxicity, and equal or higher tumor tissue concentrations than systemic chemotherapy of TPCE in two animal studies^8,9^. The effect of local tumor control and prolonged survival for pulmonary metastasis has been mentioned in combination with subsequent RFA or MWA^10–12^. However, the treatment potential for recurrent or advanced NSCLC has not yet been explored.

This study, therefore, aims to verify the potential ability of the hybrid non-surgical treatment of TPCE and MWA for the treatment of recurrent or advanced NSCLC.

## Materials and methods

### Study design

**Fig. 1:**
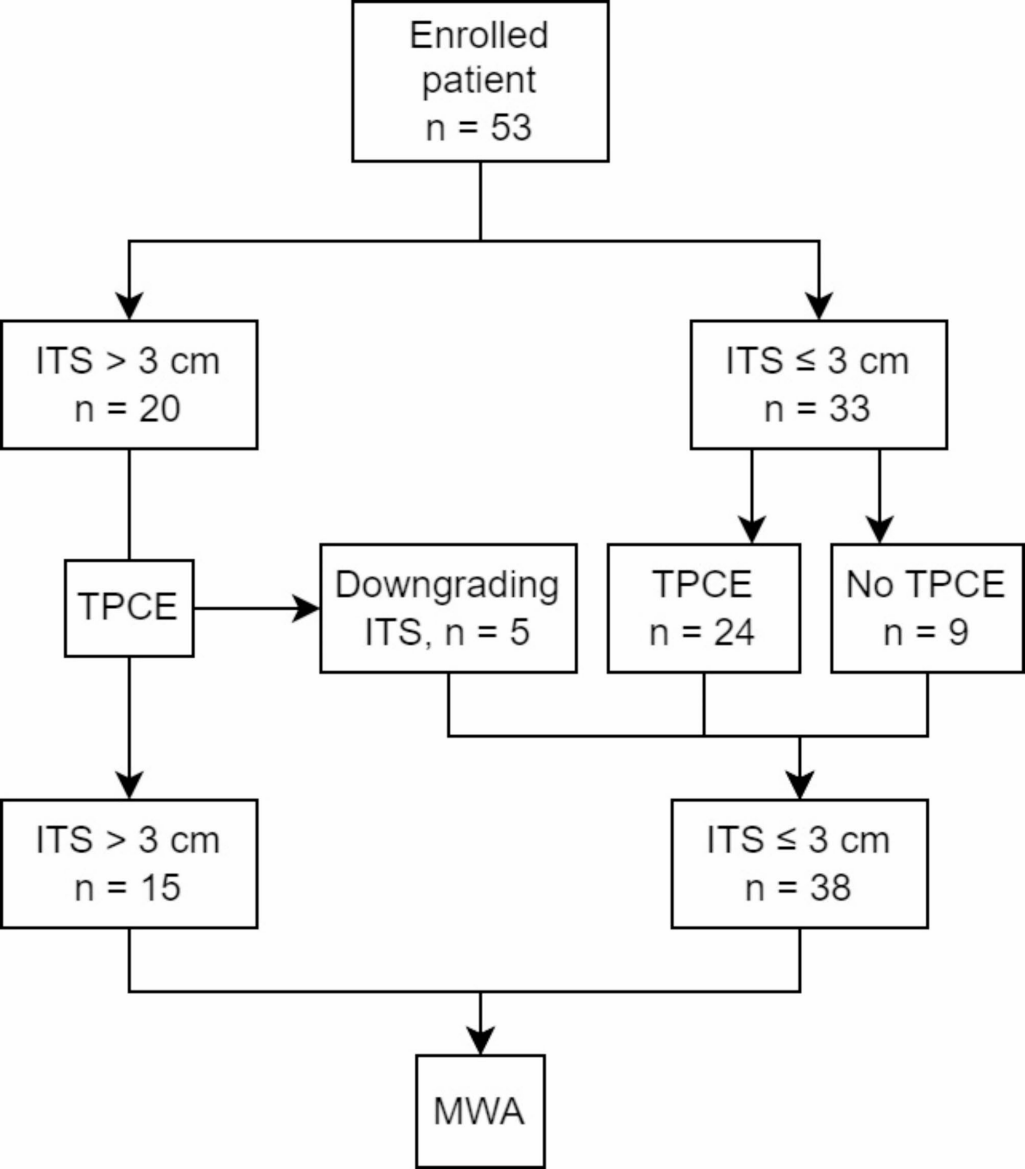
Study protocol. *ITS*- Index tumor size. *MWA*- Microwave ablation. *TPC* - Transpulmonary chemoembolization.

This retrospective single-center study was approved by the ethics committee of the Faculty of Medicine of the Goethe University Frankfurt (Approval number: 2023 − 1353). This study was conducted in accordance with the Declaration of Helsinki. The requirement to obtain informed consent was waived. Patients with non-operable NSCLC, which failed to control the local-regional tumor status under every first or second-line treatment, were recruited. A combination of TPCE and MWA or MWA alone would be applied for these patients at an affiliated academic center. Inclusion criteria were as follows: (1) patients with pathological diagnosis of NSCLC (2) patients had no more than three identifiable index tumors and a life expectancy longer than 6 months; (3) patients could lie in a prone or supine position for at least half an hour and corporate with physician or nurse. Exclusion criteria were: (1) NSCLC with a diffused local spreading disease, including multifocal tumors with more than three identifiable index tumors, pleural seeding with recognizable pleural mass, or lymphangitis carcinomatosis (2) uncorrectable coagulopathy; (3) patients who were contraindication for CT imaging or contrast medium injection; (4) presence of an acute inflammatory disease or uncontrolled systemic disease and (5) patients who refused to follow the study protocol or met the contra-indication of TPCE^7^. Due to the limited therapeutic effect of MWA in lesions with a diameter > 3 cm, indicating a higher possibility of residual lesion even under complete ablation and a higher chance of local recurrence^13^, the enrolled patients were divided into two groups based on the diameter of the largest tumor lesion, which was defined as index tumor size (ITS). Patients with ITS larger than 3 cm received several sessions of TPCE ahead of MWA according to the patient’s general condition. In this study, no more than five treatment sessions were applied. The TPCE treatment would end if the diameter of the target lesion decreased to 3 cm, the patient suffered from local tumor progression, refused to take additional TPCE, or could not apply TPCE due to their general condition. For ITS no more than 3 cm, patients were advised to take at least one session of TPCE treatment before MWA. Although, patients in this subgroup who refused TPCE therapy went directly through MWA (Fig. 1). All patients gave written informed consent before a MWA or TPCE procedure. The tumor board reviewed and adjusted all the patients’ treatment plans during the study period in our institute to optimal control their disease status.

### TPCE

The type, dose, and combination of the chemotherapeutic agents for TPCE were determined according to the conclusion of multidisciplinary tumor boards. In brief, different combinations of mitomycin C 8 mg/m^2^ (mito-medac^®^, Medac), gemcitabine 800 mg/m^2^ (Gemcitabin HEXAL^®^, Hexal AG), cisplatin 35 mg/m^2^ (Cisplatin Accord, Accord Healthcare Limited), and irinotecan 100 mg/m^2^ (Irinotecan Aurobindo^®^, PUREN Pharma GmbH) were administered^10,14^.

Before each intervention, all patients received a combination of 20 mg of dexamethasone (Fortecortin; Merck KGaA, Darmstadt, Germany), 3 mg of granisetron (Kevatril; Roche Pharma AG, Grenzach-Wyhlen, Germany), and 100 mg of pethidine (Dolantin; Sanofi-Aventis Deutschland GmbH, Frankfurt, Germany) as antiemetic and analgesic medication^15^. TPCE was performed by one physician with more than 15 years of experience using several catheters such as pigtail, headhunter and cobra catheters. An initial diagnostic pulmonary angiography was performed before cannulation of the tumor-supplying pulmonary artery branches.

After successful cannulation, the chemotherapeutic agents were injected, followed by 5–10 ml of Lipiodol^®^ (Guerbet) and 200–450 mg of degradable starch microspheres (200 µg) (EmboCept^®^, PharmaCept GmbH) as embolizers^16^. The embolization was ended when the antegrade flow was near stasis in the feeding pulmonary arteries^12^. In bilateral lung involvement, the lung with the higher tumor burden was treated initially, and the other lung was treated in successive sessions.

### MWA

MWA was performed by the same experienced physician using CT fluoroscopic guidance (Somatom Sensation 64; Siemens, Erlangen, Germany) with the following parameters: 5-mm collimation, 30 mAs, 120 kV, and 5-mm section thickness. The Emprint™ ablation system (Covidien, Mansfield, MA) was used. In every MWA session, one single antenna was used to ablate, regardless of lesion size. All ablation procedures were performed according to the manufacturer’s recommendations, including the total ablation time and the amount of energy tolerated by the patient, to achieve total coverage of the lesion into the ablation zone^17^. If the ablation zone could not cover the lesion during treatment, we would add another ablation procedure to achieve optimal treatment effect.

All patients who tolerated the ablation received analgesic medication with Morphine (10 mcg/kg/hr) during the treatment. The blood pressure and pulse oximetry were monitored continuously. The microwave antennas were inserted into the lesion under CT guidance using the trocar method. CT fluoroscopic imaging was performed every 30 s throughout the ablation procedure to assess the ablation area, location of antennas, and possible complications. At the end of each session, the needle track was coagulated to prevent the seeding of tumor cells in the needle track while removing the electrode^17,18^.

### Treatment evaluation

Routine control imaging included an unenhanced CT or MR imaging before each session and a post-interventional control unenhanced CT imaging to exclude any complications, which was done before discharging the patient who received TPCE and 24 h after an MWA session. Post-interventional control unenhanced CT imaging after the last MWA treatment would be defined as a baseline image for response evaluation. Follow-up CT or MR imaging was done to assess the therapy response 3, 6, 12, 18, and 24 months after completing all treatment sessions^10,17^.

Clinical and demographic information was extracted from the electronic clinical and imaging database. Demographic (age and gender) and tumor-specific characteristics (the number of lesions, location, pathological diagnosis, staging, and prior treatments) were then analyzed retrospectively. A researcher measured the diameter of the index lesion for the tumor size. In the case of multiple ablated lesions, the average diameter of all lesions was considered as tumor size. Outcome variables included tumor response to treatment, time to progression, survival, and complications. Treatment response was evaluated according to the revised Response Evaluation Criteria in Solid Tumors (RECIST 1.1)^19^. Complications related to TPCE or MWA were assessed according to the CIRSE classification system for complications (Table 2)^20^.

### Statistical analysis

We analyzed the data with STATA 14 for Windows (StataCorp. 2015. Stata Statistical Software: Release 14. College Station, TX: StataCorp LP.). U-test was applied to analyze complication rate and difference in two groups categorized based on the ITS, i.e., ITS ≤ 3 and > 3 cm. Survival analysis was done for all patients and the two ITS categories. The additional effect of TPCE was analyzed separately in ITS ≤ 3 cm for patients who underwent TPCE. Kaplan-Meier curve and log-rank tests were applied for overall survival^14^ and time to local tumor progression (TTP). Competing risk adjustment was applied for TTP analysis^21^ because distant tumor recurrence was beyond our study scope. The Cox-proportional hazard method was used to analyze the significance of the gathered factors. A p-value of 5% or less was considered statistically significant.

## Results

### Patient demographic

Our cohort (Table 1) included 53 patients (64.5 ± 11.4 years old), with 34 male and 19 female patients. Among all patients, 49 (92%) had adenocarcinoma. Nineteen (36%) of the patients had recurrent primary lung cancer; the remainder had advanced-stage tumors refractory to previous standard therapy.Thirty-three patients (62%) had ITS ≤ 3 cm, among which 9 (27%) were treated only with MWA. An average of 3.6 ± 2.5 chemoembolization sessions were performed on patients treated with TPCE. A total of 45 (85%) patients were treated with a single session of MWA, and the remaining nine patients underwent multiple ablation sessions. Detailed characteristics of the patients are shown in Table 2.

**Table 1:** Patient demographics.

Enrolled patients	*n* = 53
**Age**	64.5 (11.4)
**Gender**	
male	34
female	19
**Pathology**	
Adenocarcinoma	49
Squamous cell carcinoma	3
Other	1
**Staging**	
I	8
II	6
III	11
IV	28
**Laterality**	
Unilateral	44
Bilateral	9
**Indication**	
Recurrent	19
Advanced	34

**Table 2:** Immediate treatment effects and complications.

**Treatment parameters**	
ITS > 3 cm	*n* = 20
ITS ≤ 3 cm	*n* = 33
**TPCE**	
With TPCE	44
Without TPCE	9
Average TPCE times	3.6 (2.5)
Index tumor size (cm)	3.07 (1.97)
Index tumor size reduction (cm) after TPCE	0.91(0.61)
Pulmonary nodule number (per patient)	1.99 (1.77)
Treatment response	
PR	12 (27%)
SD	32 (73%)
Complication (TPCE)	8 (18%)
Grade l	7 (16%)
Grade 3	1 (2%)
**MWA**	
Single section	45
Multiple sections	8
Complication (MWA)	25 (47%)
Grade 1	17 (32%)
Grade 3	8 (15%)

*ITS*- Index tumor size.

*MWA*- Microwave ablation.

*PR- *Partial regression.

*SD- *Stable disease.

*TPCE*- Transpulmonary chemoembolization.

### Treatment effect

#### Immediate effect and complications

**Fig. 2:**
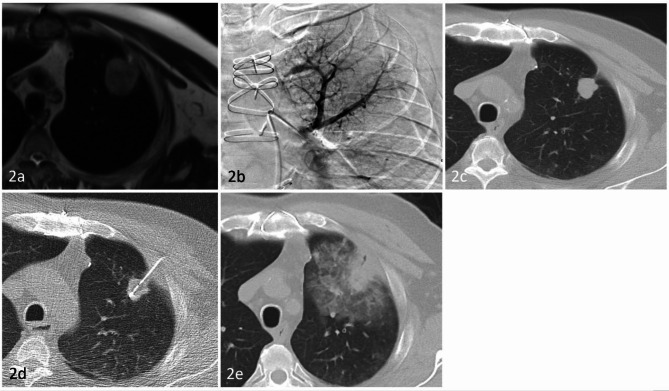
A 66 Y/O male suffered from LUL NSCLC. MR image before TPCE (2a) showed a LUL mass (4.3 cm). First TPCE was performed (2b) smoothly. After 3 sessions of TPCE. CT image (2c) showed size decreasing (4.3 to 2.3 cm) of the index lesion. MWA was performed subsequently (2d). CT image (2e) after MWA. ITS - Index tumor size. LUL- Left upper lung. NSCLC - Non-small cell lung cancer. MWA - Microwave ablation. TPCE - Transpulmonary chemoembolization.

Overall treatment response in our study after TPCE showed 12 patients (27%) with PR status and 32 patients (73%) with SD status. There was no patient with PD status in our case series before MWA. An average tumor size reduction of 0.91 ± 0.61 cm was shown in patients treated with TPCE (Fig. 2). All patients tolerated the TPCE procedure, with seven suffering from grade 1 complications, including transient fever and chest tightness. One patient had a grade 3 complication as bronchospasm due to an allergic reaction related to cisplatin and was treated successfully under steroid injection.

All the index tumors ablated smoothly in our study. After ablation, the total complication rate was 47%. 32% (17/53) of the patients suffered grade 1 complications, including pneumothorax or intrapulmonary hemorrhage, and 15% (8/53) of patients presented with grade 3 complications, including six patients with pneumothorax needing further drainage and two with hemothorax requiring additional embolization/ surgery. There was no procedure-related mortality in this study. The complication rate of MWA was significantly higher in ITS ≤ 3 cm than in ITS > 3 cm (*p* = 0.013). Seven out of the eight occurred major complications were in ITS ≤ 3 cm group (Table 3).

**Table 3:**
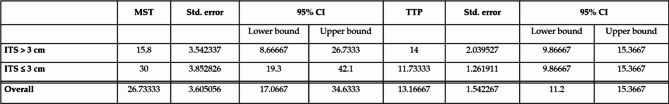
MST and TTP according to ITS group before MWA.

#### Survival analysis

Our patients’ median survival time (MST) was 26.7 (15.3–38.8) months. By Kaplan–Meier analysis, the overall survival rates at one year, two years, and four years were 81.2%, 53.3%, and 18.8%, respectively. The ITS ≤ 3 cm group patients had better survival outcomes than the others (MST: 31.6 vs. 15.8 months, *p* = 0.003) (Table 4). Patients with recurrent tumors had better MST than patients with advanced lung cancer without significant difference (30 vs. 15.7 months, *p* = 0.08). Prognostic factors of overall survival by univariate Cox-proportional hazard regression models was ITS > 3 cm (HR: 1.18, *p* = 0.02). Other factors such as age, gender, pathology, tumor staging, total lesion number, and unilateral or bilateral distribution of the lesion were not significant. After competing risk adjustment, the TTP of all patients was 13.2 months, with only the bilateral distribution of the target lesions demonstrating significant survival differences in unilateral distribution (8.6 vs. 22.8 months, *p* = 0.0016) (HR: 2.66, *p* = 0.02).

**Table 4:** Subgroup analysis of patients treated with TPCE.

Subgroup with TPCE (*n* = 44)			
	ITS > 3 cm	ITS ≤ 3 cm	P value
**Before TPCE**	*n* = 19	*n* = 25	
Age	66.81 (8.99)	62.79 (10.99)	0.21
Gender	M: 9/F: 10	M: 19/F: 6	0.09
Standard treatment before enrollment	9/19 (47%)	18/25 (72%)	0.61
Pathology			0.14
Adenocarcinoma	19	21	
Squamous cell carcinoma	0	3	
Other	0	1	
Staging			0.48
I	2	3	
II	2	4	
III	6	3	
IV	9	15	
TPCE times	4.87 (1.45)	3.96 (2.33)	0.17
Laterality			0.87
Unilateral	17	21	
Bilateral	2	4	
**After TPCE**	*n* = 15	*n* = 29	
Index tumor size reduction (cm)	1.06 (0.16)	0.44 (0.11)	0.004
MWA times	1.06 (0.25)	1.18 (0.39)	0.29
Complication (TPCE)	3/15(20%)	5/29 (17%)	0.31
Complication (MWA)	3/15 (20%)	20/29 (59%)	0.004
**ITS > 3 cm after TPCE**			
**Group shifting**	**No** (*n* **= 15**)	**Yes** (*n* **= 4**)	
Treatment response (numder of PR)	*n* = 2 (13%)	*n* = 3 (75%)	0.000
MST	15.8 month	22.2 month	0.06

*ITS*- Index tumor size.

MST-Mean survival time.

*MWA*- Microwave ablation.

PR- Partial regression.

*TPCE*- Transpulmonary chemoembolization.

### Subgroup analysis for all patients treated with TPCE

A significantly better PR rate (34% vs. 13%, *p* = 0.04) was noted in ITS ≤ 3 cm than in ITS > 3 cm. There was no significant difference in pre-treatment parameters, total sessions of TPCE, and total tumor numbers before and after TPCE between the two ITS groups. 21% (4/19) of the patients shifted from ITS > 3 cm to ITS ≤ 3 cm after TPCE treatment, which demonstrated a significantly better PR rate (75% vs. 13%, *p* = 0.00) than those remaining in ITS > 3 cm; however, only borderline significant better MST was demonstrated in this subgroup than those remaining in ITS > 3 cm (22.2 vs. 15.8 months, *p* = 0.06). Before the study ended, no mortality or local recurrence was noted in the ITS-shifting patient group. The effect of index tumor size reduction was significantly higher in ITS > 3 cm than in ITS ≤ 3 cm (1.06 ± 0.16 vs. 0.44 ± 0.11, *p* = 0.004). After MWA, the complication rate is significantly lower in ITS > 3 cm than in ITS ≤ 3 cm (20% vs. 69%, *p* = 0.004). The MST was significantly lower in ITS > 3 cm (15.8 vs. 23.1 months, *p* = 0.039), and a significantly lower survival benefit (HR:2.24, *p* = 0.044) was also noted.

### Comparing TPCE impact in ITS ≤ 3 cm group

Patients originally in this group who underwent TPCE had lower MST than those who were treated with MWA alone without any statistical significance (23.1 vs. 38 months, *p* = 0.08). However, the complication rate was significantly higher in patients treated with TPCE than those without TPCE (69% vs. 22%, *p* = 0.007).

## Discussion

Our study showed significant survival benefits for patients with ITS ≤ 3 cm, which aligns with previous studies^5,22^. However, the therapeutic benefit of MWA was limited for lesions with ITS > 3 cm^13^. A likely explanation would be a reduced probability of achieving complete ablation for index tumors^13,23^. In index tumors with unilateral lung distribution, significantly higher TTP than in bilateral lung distribution, which was only reported in a previous study with better MST for pulmonary metastasis^10^. This feature may relate to the difference in pathophysiology between NSCLC and pulmonary metastasis and the additional treatment effect of TPCE.

There were no severe complications in patients receiving TACE. Similar to previous publications, it confirms that TPCE is a safe procedure^10,15^. The complication rate was significantly higher in patients within ITS ≤ 3 cm and in tumors with complete ablation, contrary to previous studies that demonstrated an increase in complication rate with increasing index tumor size^13,24,25^. The evolution of the MWA systems from 915 MHz to 2450 MHz^26,27^, with much higher heating power during ablation, might be a possible reason.

TPCE served as an effective method for temporary disease control in this study. No patient suffered from disease progression in our study before MWA, with significantly more PR cases in the ITS ≤ 3 cm group. It also demonstrated an additive effect on decreasing tumor size, which seems more prominent in large tumors. 21% of the included patients in ITS > 3 cm shifted to ITS ≤ 3 cm and showed survival benefits compared to those who remained in ITS > 3 cm with borderline significance. No mortality or local recurrence was noted in the ITS-shifting patient group before the study ended, but the small number of these patients limited the interpretation of this phenomenon. However, TPCE had no survival benefit for patients in ITS ≤ 3 cm, comparing those who were not treated with TPCE. Patients treated with TPCE in the ITS ≤ 3 cm group had significantly higher complication rates after MWA than the patients in the same group treated with MWA alone.

MWA is a rapidly emerging treatment for lung tumors. Like other thermal ablation techniques, such as RFA and cryoablation, it could be an alternative option for curative therapy in patients with localized NSCLC who cannot be treated by surgery or SABR^28^. However, more extensive evidence is still needed when considering more complex scenarios, such as tumor recurrence after surgery or non-operable advanced NSCLC.

Our study demonstrated non-significant higher MST for recurrent NSCLC (30 months) than advanced NSCLC refractory to standard treatment (15.7 months). A recent study showed the treatment effect of MWA on oligo-recurrence with primary stage I-III NSCLC with MST of 40.6 months^29^, which was marginally higher than our results. There are few similar studies on advanced NSCLC with limited case numbers, such as combination treatment of MWA with chemotherapy with MST of 9.6 months^30^ or MWA with an immune checkpoint inhibitor in which MST was not reached before the end of the study^31^. Due to the heterogeneity of the patient condition in advanced NSCLC, further study of alternative treatment should be cautious on patient selection to evaluate the treatment response precisely.

Our study had several limitations: retrospective design and small case numbers, which resulted in insufficient power for subgroup analysis. The complexity of the patient source from different hospitals and countries hindered the precise acquirement of previous treatment details, which might influence the treatment outcome of our study. The lack of a control group in ITS > 3 cm caused a suboptimal analysis of the actual treatment of TPCE combined with MWA. A considerable amount of missing documented data was confronted during the study, leading to difficulties in the adjustment of detailed medical history, the actual date of disease progression, and treatment details after the progression.

## Conclusion

In conclusion, TPCE effectively controlled the tumor progression in our study, which might be a potential bridging treatment for patients waiting for more treatment options. Combining TPCE and MWA might improve local tumor control and prolong survival for recurrent or advanced NSCLC with ITS > 3 cm, mainly because of decreasing tumor size after TPCE. For ITS ≤ 3 cm, due to the lack of direct evidence of better treatment effect in TPCE with MWA than MWA alone, caution should be taken when considering treatment options for a patient. Further investigation is warranted to explore the effect of combination treatment and its role in recurrent and advanced NSCLC.

## Data Availability

The datasets generated and/or analysed during the current study are not publicly available due to privacy but are available from the corresponding author on reasonable request.
